# Anxiety disorders and self-esteem levels of primary school children

**DOI:** 10.1192/j.eurpsy.2025.1186

**Published:** 2025-08-26

**Authors:** F. Erotokritou, I. Koutelekos, A. Soldatou, A. Zartaloudi

**Affiliations:** 1 MSc in General Pediatrics and Pediatric Subspecialties - Clinical Practice and Research, University of Athens; 2 University of West Attica; 3 University of Athens, Athens, Greece

## Abstract

**Introduction:**

There is no doubt that childhood is a crucial period in a person’s development. Anxiety disorders in modern times are at the forefront, with a huge number of children and adolescents suffering from them. An important asset, a protective shield against anxiety disorders that promotes mental well-being is self-esteem.

**Objectives:**

The present study aims to investigate the level of self-esteem and the differentiation of various anxiety disorders, as well as the assessment of the severity of anxiety symptoms exhibited by children aged 9-12 years, who were attending the 4th, 5th, and 6th grades of public primary schools in the Larnaca district of Cyprus. Additional objectives are to investigate possible associations between these variables and the socio-demographic characteristics of the students in combination with their teacher-reported school performance.

**Methods:**

To 249 students were administered (a) a Sociodemographic Characteristics Questionnaire, (b) the Rosenberg Self-Esteem Scale, (c) the Spence Children’s Anxiety Scale (SCAS), and their teachers were given a questionnaire to assess the academic performance of each student individually.

**Results:**

The protective role of self-esteem in the development of anxiety disorders (p<0.001) and feelings of loneliness and the positive effect on school performance was confirmed by the study. Besides, high levels of self-esteem were associated with reduced feelings of loneliness (p 0.001), increased school performance (p 0.020) and greater satisfaction with teacher help (p 0.001). Additionally girls had more frequent anxiety disorder behaviours compared to boys (p 0.028), especially panic disorders, agoraphobia (p 0.020), specific phobias (p 0.012) and separation anxiety (p 0.005). Interestingly, the study found that stronger feelings of loneliness at school were associated with increased occurrence of anxiety disorders (p<0.001). It was also found that older students were less likely to develop anxiety disorder behaviours (p 0.002), while children with more siblings in a family showed worse ratings of student performance at school (p 0.001). Finally, it appears that school performance was associated with less social phobias (p 0.048) and that those children who received parallel support had lower performance than those who did not (p<0.001).

**Conclusions:**

Promoting increased levels of self-esteem, especially in children, is very important as it can help safeguard mental health. Anxiety disorders are a crucial problem in modern times, so their continuous study and treatment are essential. Holding informational and awareness-raising conferences for teachers, parents, and generally all professionals who come into frequent contact with children at this sensitive age is necessary and indisputable.

**Disclosure of Interest:**

None Declared

